# *ESR1* Mutations Are Not a Common Mechanism of Endocrine Resistance in Patients With Estrogen Receptor–Positive Breast Cancer Treated With Neoadjuvant Aromatase Inhibitor Therapy

**DOI:** 10.3389/fonc.2020.00342

**Published:** 2020-04-03

**Authors:** Tomás Reinert, Susana Ramalho, Vivian Castro Antunes de Vasconcelos, Leonardo Roberto Silva, Ana Elisa Ribeiro da Silva, Camila Annicchino de Andrade, Maria Beatriz de Paula Leite Kraft, Guilherme Portela Coelho, Jovana Mandelli, Monique Binotto, Cesar Cabello, Geisilene Russano de Paiva Silva, José Bines, Carlos H. Barrios, Matthew J. Ellis, Marcia Silveira Graudenz

**Affiliations:** ^1^Postgraduate Program in Medical Sciences, Universidade Federal Do Rio Grande Do Sul (UFRGS), Porto Alegre, Brazil; ^2^Centro de Pesquisa da Serra Gaucha (CEPESG), Caxias Do Sul, Brazil; ^3^Latin American Cooperative Oncology Group (LACOG), Porto Alegre, Brazil; ^4^Department of Obstetrics and Gynecology, Faculty of Medical Sciences, State University of Campinas (UNICAMP), Campinas, Brazil; ^5^Diagnose Patologia e Biologia Molecular, Caxias Do Sul, Brazil; ^6^Laboratory of Molecular and Investigative Pathology – LAPE, Faculty of Medical Sciences, State University of Campinas (UNICAMP), Campinas, Brazil; ^7^Instituto Nacional Do Câncer (INCA – HCIII), Rio de Janeiro, Brazil; ^8^Lester and Sue Smith Breast Cancer Center, Baylor College of Medicine, Houston, TX, United States

**Keywords:** breast cancer, endocrine therapy, *ESR1*, *ESR1* mutations, neoadjuvant, aromatase inhibitors

## Abstract

**Introduction:** Mutations in the *ESR1* gene (*ESR1*m) are important mechanisms of resistance to endocrine therapy in estrogen receptor–positive (ER+) metastatic breast cancer and have been studied as a potential therapeutic target, as well as a predictive and prognostic biomarker. Nonetheless, the role of *ESR1*m as a possible mechanism of primary endocrine resistance, as well as whether it also occurs in tumors that are resistant to ET administered in early-stage disease as (neo)adjuvant, has not been adequately studied. In this study, we evaluated the prevalence of *ESR1*m in tumor samples from patients with ER+ breast cancer resistant to neoadjuvant aromatase inhibitor therapy.

**Methods:** We followed a prospective cohort of patients with ER+ HER2– stages II and III breast cancer treated with neoadjuvant endocrine therapy (NET). Tumor samples from patients with a pattern of primary endocrine resistance [defined as a Preoperative Endocrine Prognostic Index (PEPI) score of ≥4] were identified and analyzed for the presence of *ESR1*m.

**Results:** One hundred twenty-seven patients were included in the cohort, of which 100 (79%) had completed NET and underwent surgery. Among these patients, the PEPI score ranged from 0 to 3 in 70% (70/100), whereas 30% (30/100) had a PEPI score of 4 or more. Twenty-three of these patients were included in the analysis. *ESR1* mutations were not identified in any of the 23 patients with early-stage ER+ breast cancer resistant to NET.

**Discussion:** Growing evidence supports the notion that there are different mechanisms for primary and secondary endocrine resistance. Our study suggests that *ESR1* mutations do not evolve rapidly and do not represent a common mechanism of primary endocrine resistance in the neoadjuvant setting. Therefore, *ESR1*m should be considered a mechanism of acquired endocrine resistance in the context of advanced disease. Further research should be conducted to identify factors associated with intrinsic resistance to ET.

## Introduction

Estrogen receptor–positive (ER+) breast cancer is the most prevalent breast cancer subtype. Endocrine therapy (ET) remains the mainstay of treatment in all stages of the disease (1). Nevertheless, endocrine resistance associated with disease progression remains an important challenge (2, 3). Mutations of the *ESR1* gene, which encodes the ER protein, have been increasingly identified as one of the most important mechanisms of endocrine resistance (4).

Breast tumors are known to undergo genomic evolution, and *ESR1* mutations (*ESR1*m) have been identified in 9–40% of patients with metastatic ER+ breast cancer resistant to aromatase inhibitors (AIs) (3–7). In Brazilian patients with visceral metastasis of ER+ HER2-negative breast cancer, our group reported the presence of *ESR1*m in 25% of the cases (8). In the metastatic setting, the presence of *ESR1*m is a biomarker of a worse prognosis. The function of *ESR1*m as a potential therapeutic target, as well as a predictive and prognostic biomarker, is being studied (9). However, the role of *ESR1*m as a possible mechanism of ET resistance in the early-stage disease is unclear. Regardless of the extensive research that is being conducted in this field, several questions remain unanswered about *ESR1*m, such as whether it is associated with endocrine resistance to AIs used with a curative intent treatment in the (neo)adjuvant setting.

Neoadjuvant endocrine therapy (NET) is a therapeutic approach that is being increasingly explored, not only to allow less extensive surgery but also as a scientific tool (10). The Preoperative Endocrine Prognostic Index (PEPI) is a surrogate of endocrine sensitivity and identifies a subgroup of patients with primary resistance to NET (11, 12).

The objective of this study was to evaluate the potential role of *ESR1*m as a mechanism of resistance in postmenopausal patients with breast cancer treated with NET with a high PEPI score as a surrogate for primarily endocrine-resistant biology.

## Methods

We conducted a prospective cohort of patients with breast cancer treated with NET in two institutions. The population included postmenopausal women presenting with stages II and III ER+ Her2-negative breast cancer. Protocols of diagnosis, therapies, and follow-up of patients were standardized and based on major international guidelines. These procedures are not part of the study.

Eligible patients were treated with NET with anastrozole for a recommended period of at least 3 months, followed by surgery. Pathological and immunohistochemistry (IHC) protocols were standardized and followed American Society of Clinical Oncology/College of American Pathologists guidelines and the international consensus of pathologic assessment of the breast and axilla after preoperative therapy (13). For all patients who underwent surgery, the PEPI score was calculated (described in Table 1). All IHC assessments were reviewed by two pathologists. Tumor samples from patients with a pattern of primary endocrine-resistant tumors (defined as a PEPI score of ≥4) were selected and analyzed for the presence of *ESR1*m.

**Table 1:** The preoperative endocrine prognostic index (PEPI) score following 4–6 months of neoadjuvant AI or other endocrine therapy provides another strategy to identify endocrine-sensitive vs. endocrine-resistant tumors in the early-stage setting.

**Surgical factors**	**RFS HR**	**PEPI score**
**TUMOR SIZE**		
T1/T2	–	0
T3/T4	2.8	3
**NODE STATUS**		
Negative	–	0
Positive	3.2	3
**Ki67 LEVEL**		
0–2.7%	**–**	**0**
>2.7–7.3%	1.3	1
>7.3–19.7%	1.7	1
>19.7–53.1%	2.2	2
>53.1%	2.9	3
**ER STATUS**		
Negative	2.8	3
Positive	0	0

*A PEPI score of 0 [pT1/2, node-negative (N0), Ki67 <2.7%, estrogen receptor-positive (ER^+^)] is being investigated prospectively as a surrogate of endocrine therapy-sensitive disease that does not need chemotherapy, and a PEPI of >0 identifies patients with an increased risk of relapse. The hazard ratio (HR) of each surgical factor for relapse-free survival (RFS) and assigned PEPI points based on the data from the P024 trial are shown in the table. Adapted from Ma et al. (3)*.

*ESR1*m were evaluated in formalin-fixed paraffin-embedded breast cancer tissue using real-time quantitative polymerase chain reaction (RT-qPCR). For each sample, the tumor area was identified and marked by the same pathologist, and this was followed by DNA extraction with the Wizard^©^ Genomic DNA Purification kit (Promega, Madison, WI, USA). DNA was quantified using Qubit Fluorometric Quantitation (Thermo Fisher Scientific, Waltham, MA, USA), and 20 ng/μL was the threshold for the analysis of the mutation. The reactions were performed with the equipment 7500 Fast Real-Time PCR System using TaqMan Genotyping Master Mix, primers, and TaqMan^©^ probes and followed all recommendations of the manufacturer. A TaqMan^©^ reference probe was used to evaluate the presence of the mutations, followed by analysis in the 7500 Software v2.06 (Thermo Fisher Scientific). The analyzed mutations were Y537N, Y537C, Y537S, E380Q, and D538G. A description of the primer sequences used for RT-PCR can be found in Supplementary Material.

Statistical analysis was performed using Statistical Package for Social Sciences 22.0 (SPSS, Armonk, NY, USA). Categorical variables are described by frequencies and percentages. The primary endpoint is the prevalence of *ESR1*m. For the primary endpoint, the point estimate is presented together with the exact 95% confidence interval. Continuous variables are described by means and standard deviation or median, depending on Shapiro–Wilk test analysis. Correlations between categorical variables were analyzed using the χ^2^ test. Differences between means are analyzed using the Student *t*-test. This project was reviewed and approved by the institutional review board (ethical committee) of both institutions.

## Results

**Figure 1:**
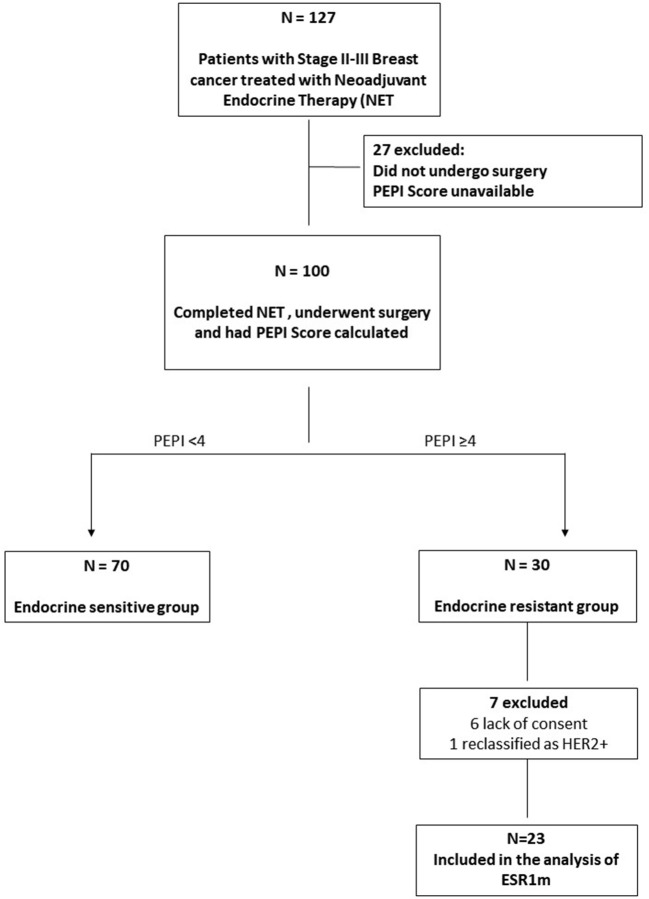
Flowchart of patients with ER+ HER2− stages II and III breast cancer treated with neoadjuvant endocrine therapy (NET). Tumor samples from patients with a pattern of primary endocrine resistance [defined as a Preoperative Endocrine Prognostic Index (PEPI) score of 4] were identified and analyzed for the presence of *ESR1*m.

One hundred twenty-seven patients were included in the cohort, of which 100 (79%) completed NET and surgery. Among these patients, the PEPI score ranged from 0 to 3 in 70% (70/100), and 30% (30/100) had a PEPI score of 4 or more and were selected. Twenty-three patients were included in the analysis (six did not consent or were lost to follow-up, and one was found to be HER2-positive in the surgical sample). These data are summarized in Figure 1.

Table 2 summarizes the most important characteristics of the patients and biopsy findings at the initial diagnosis. Tumors classified as primarily resistant to ET (PEPI score of four or more) were selected and had their tumors analyzed for *ESR1*m. In our analysis, the median age was 70 years. There was a predominance of ductal carcinoma (86.9%) and T1–T2 tumors (60.8%), and the majority of our patients had clinical positive axillary nodes (56.5%) before treatment and had a Ki67 index between 7.4 and 19.7%. Table 3 describes the results in terms of PEPI score and pathological and IHC evaluation. On pathological analysis (after NET), most of the tumors were T1/T2 (73.9%), had a compromised axilla (91.3%), maintained ER positivity (95.6%), and had intermediate levels of Ki67.

**Table 2:** Patients characteristics, pathology and IHC results (initial biopsy sample) (*n* = 23).

**Age**	**Median 70 years (54–84)**
Histology	Ductal: 20 (86.9%) Lobular: 3 (13.1%)
Clinical tumor size	cT1/T2: 14 (60.8%) cT3/T4: 9 (39.2%)
Clinical lymph node status	Negative (cN–): 10 (43.4%) Positive (cN+): 13 (56.5%)
Ki67 level—baseline	<2.7%: 2 (8.6%) 2.8–7.3%: 1 (4.3%) 7.4–19.7%: 12 (52.1%) 19.8–53.1%: 8 (34.7%) >53.2%: 0
Duration of NET	Median 22 weeks (4–35)

**Table 3:** PEPI Score, pathology and IHC results after NET (surgical sample).

PEPI score	4: 16 (69.5%) 5: 2 (8.6%) 6: 3 (13%) 7: 2 (8.6%)
Pathological tumor size—surgical specimen	ypT1/T2: 17 (73.9%) ypT3/T4: 6 (26%)
Pathological lymph node status	Negative (ypN–): 2 (8.6%) Positive (ypN+): 21 (91.3%)
ER status, Allred score	0–2: 1 (4.3%) 3–8: 22 (95.6%)
Ki67 level—surgical specimen	<2.7%: 5 (21.7%) 2.8–7.3%: 5 (21.7%) 7.4–19.7%: 11 (47.8%) 19.8–53.1%: 1 (4.3%) >53.2%: 1 (4.3%)

The median duration of NET was 22 weeks (range, 4–35 weeks). All samples of tumor tissue from the surgical specimens after NET were evaluable. Quantification of DNA extraction and reference gene cycle threshold values confirmed that the material was adequate for the analysis. *ESR1* mutations were not identified in any of the 23 samples. Therefore, the prevalence of *ESR1*m in patients with early-stage ER+ HER2-negative breast cancer that were intrinsically resistant to NET is 0% (95% confidence interval, 0–12.2%).

## Discussion

Our study reports that differently than in AI-resistant metastatic breast cancer, *ESR1*m are not a common mechanism of resistance in tumors with primary endocrine resistance in the neoadjuvant setting. This finding may have implications for the future development of *ESR1*m as a biomarker and therapeutic target.

Estrogen receptor–positive tumors are the most common form of breast cancer and the major cause of mortality by this disease worldwide (14). The use of endocrine agents that interfere with the ER pathway such as tamoxifen and AIs remains the standard-of-care treatment both for patients with early-stage disease and for advanced disease when the use of ET is associated with objective response and disease control in a significant proportion of patients. Nevertheless, tumor evolution caused by a complex network of mechanisms of endocrine resistance continues to be an important challenge (14). In early-stage disease, a considerable proportion of patients will develop disease recurrence despite the use of curative-intent treatment with a combination of therapies such as surgery, chemotherapy, radiation, and adjuvant hormone therapy.

Estrogen receptor, a protein encoded by the *ESR1* gene, is expressed in the majority of breast cancers and is one of the key factors for disease classification and treatment definition. A significant body of research has confirmed the essential role of the ER pathway in physiological mammary gland development and also in tumorigenesis (15). Estrogen binding triggers several events, resulting in conformational changes in the ligand-binding domain (LBD), receptor activation, and allowing the ligand–receptor complex to bind to specific sequences of the genome and to interact with corepressor and coactivator proteins to regulate the transcription of estrogen-response elements (16). The most common molecular alterations in the *ESR1* gene found in breast tumors are mutations, most commonly missense mutations found in codons 380, 537, and 538. *ESR1*m are most commonly missense mutations clustered in codons 537 and 538 of the LBD (17). The most prevalent *ESR1* point mutations are Y537S and D538G, whereas others have been described at significantly lower frequencies (15). Additionally, *ESR1* is known to undergo amplifications and translocations that are potential mechanisms of resistance to ET (17–19).

*ESR1*m have been consistently associated with more aggressive disease, presence of visceral metastasis, and worst prognosis (8, 15, 20). Extensive research addressing the potential role of *ESR1*m as a predictive biomarker to guide therapeutic decisions is ongoing (20). Additionally, the development of targeted therapies directed to tumor cells harboring *ESR1* mutations is a logical and appealing concept, and extensive preclinical research has been conducted in this field (4, 21), and promising work evaluating treatment with new generation endocrine agents is in progress (22, 23).

The role of *ESR1*m has been mostly studied in the metastatic setting in patients who had disease progression on first-line ET, usually presenting with a clinical pattern of acquired (secondary) ET resistance. Nevertheless, the potential role of *ESR1*m in patients with early-stage breast cancer treated with (neo)adjuvant ET has not been adequately studied. While *ESR1*m have been identified in advanced breast cancer, their presence in primary tumors is very low (24). Understanding mechanisms of intrinsic (acquired) endocrine resistance is of great importance, given that it could lead to the development of biomarkers and therapeutic agents that could be incorporated in the context of curative-intent treatment of breast cancer.

Historically, NET has been indicated as a clinical tool for tumor downstaging to allow breast-conserving surgery. Recently, NET is being used not only as a clinical tool but also as a scientific tool for the study of tumoral patterns of intrinsic endocrine sensitivity or resistance. Importantly, NET allows an *in vivo* observation of the response to estrogen deprivation therapies (25).

Preoperative Endocrine Prognostic Index, described in Figure 1, was created to distinguish tumors that are sensitive to NET from tumors that have an intrinsic degree of endocrine resistance (25). This tool is composed of four independent prognostic factors: pathological tumor size, lymph node status, level of ER expression, and Ki67 expression at the surgical sample following neoadjuvant AI therapy. The PEPI score was validated in samples from patients included in two NET clinical trials: the PO24 and IMPACT studies (11). In both trials, the rate of early relapse was very low in patients with a PEPI score of zero (25). The rate of PEPI-0 tumors in neoadjuvant AI therapy trials ranges from 17 to 37% (25). This population has a very favorable prognosis, and these patients could potentially receive adjuvant endocrine monotherapy and be spared from chemotherapy (3). Preoperative Endocrine Prognostic Index scores greater than zero were associated with an incremental increase in the risk of relapse, especially in patients with a score higher than 3 (12).

These findings were compared with a study from our group using the same methodology in patients with advanced disease where *ESR1* mutations were identified in 25% (*n* = 32) of cases with visceral metastasis of ER+ breast cancer resistant to ET (8). A statistically significant association of the presence of *ESR1*m in metastatic disease compared with tumors resistant to AIs used in the neoadjuvant setting was demonstrated (*p* = 0.01, Fisher exact test).

Therefore, the discovery of biomarkers associated with resistance to NET, as well as adjuvant ET, remains an unmet need. Our data reinforce the notion that mutations in the *ESR1* gene do not seem to evolve rapidly and are probably mechanisms of secondary resistance to ET. Ongoing studies are evaluating a variety of potential mechanisms of primary endocrine resistance, such as defects in the DNA repair machinery (26) and aberrant FGFR signaling (27).

Our study has limitations, and we understand that the detected prevalence of *ESR1*m can be underestimated given the fact that a PCR-based methodology analyzed only specific mutations in the most commonly mutated codons. Therefore, it is possible that cases with mutations in different codons of the *ESR1* gene potentially detectable with next-generation sequencing (NGS) technologies were not identified (28, 29). However, it is essential to emphasize that a robust body of literature clearly shows that *ESR1*m usually occurs in hotspots within the LBD, and the vast majority of the published data in this field reported mutations in the same codons we studied (15). Moreover, other studies showed that a specific real-time PCR assay does provide a rapid and reliable diagnostic tool for accurate detection of *ESR1*m (30).

We acknowledge that unidimensional biomarker analysis is not the best approach to investigate primary resistance and that NGS-based evaluation of whole-genome sequences, whole-exome sequencing, and even targeted gene panels could expand the identification of mechanisms of endocrine resistance. Nevertheless, the evaluation of the role of other mutations associated with endocrine resistance is beyond the scope of this article.

Additionally, and despite the challenge of conducting a prospective cohort of NET that includes more than 100 breast cancer patients, we acknowledge that the relatively low sample size of 23 cases of tumors with a PEPI score of 4 or more is a limitation and highlights the challenges of studying primary endocrine resistance. Furthermore, our patients were treated with a median 3 months of NET. Therefore, we cannot exclude that resistance documented after longer treatment durations (6–12 months) could not be related to receptor mutations. Still, in these cases, it would be expected that patients would have at least stable disease or a degree of response to allow for the continuation of treatment. These considerations would make it unlikely to relate *ESR1*m with primary resistance.

Among the strengths of our study, we highlight the conduction of a well-designed prospective cohort including 127 breast cancer patients who were treated with standard-of-care NET and whose tumors were evaluated with uniform procedures of pathology and IHC. Additionally, we used a validated methodology for *ESR1* determination, and we were able to perform a prespecified comparison in two different settings (neoadjuvant and metastatic). Despite the relatively low sample size, we demonstrated that the prevalence of *ESR1*m in NET-resistant tumors is very low (95% of chance of being inferior to 12%). Therefore, the chances that this molecular alteration will have a practical role for patient care in this setting are minimal.

Integrative approaches to evaluate the genetic and phenotypic heterogeneity of breast tumors with the use of a more comprehensive analysis of the genome, transcriptome, epigenetic regulators, and modern quantitative proteomics methods associated with advanced bioinformatics and statistical analysis may lead to advances that can potentially be translated into improved outcomes for cancer patients (31). Advances in the understanding of the molecular biology of ER+ breast cancer led to a revolution in this field with the development of a variety of therapeutic agents that are now being used in routine clinical care in patients with metastatic disease such as CDK4/6, PI3K, and mTOR inhibitors (1). However, a clinically useful biomarker to identify primary sensitivity and resistance to ET has not yet been developed and remains an unmet need.

## Conclusion

Growing evidence supports the notion that there are different mechanisms of primary and secondary endocrine resistance. Our study suggests that *ESR1* mutations do not evolve rapidly and do not represent a common mechanism of primary endocrine resistance in the neoadjuvant setting. Therefore, *ESR1*m should be considered a mechanism of acquired endocrine resistance in the context of advanced breast cancer. Further research should be conducted to identify factors associated with intrinsic resistance to ET.

## Data Availability Statement

All datasets generated for this study are included in the article/Supplementary Material.

## Ethics Statement

All procedures performed in studies involving human participants were in accordance with the ethical standards of the institutional and/or national research committee and with the 1964 Helsinki declaration and its later amendments or comparable ethical standards. Informed consent was obtained from all individual participants included in the study. This article does not contain any studies with animals performed by any of the authors.

## Author Contributions

TR, SR, CC, JB, CB, ME, and MG designed the study. TR, SR, VV, LS, CC, CA, AS, and MK were involved in the oncologic treatment of patients. GC, JM, GP, and MG did the pathologic and molecular biology evaluation. TR, SR, VV, and MB analyzed and interpreted the data. All authors read and approved the final manuscript.

### Conflict of Interest

TR received research funding from AstraZeneca and speaker honoraria from AstraZeneca, Lilly, Novartis, Pfizer, and PierreFabre. CB received research funding, speaker honoraria and participated in advisory boards from Roche, Pfizer, GSK, Boehringer Ingelhein, Eisai, AstraZeneca, BMS, MSD, Libbs. The remaining authors declare that the research was conducted in the absence of any commercial or financial relationships that could be construed as a potential conflict of interest.
