# Supplementary material for: ESR1 Mutations Are Not a Common Mechanism of Endocrine Resistance in Patients With Estrogen Receptor–Positive Breast Cancer Treated With Neoadjuvant Aromatase Inhibitor Therapy
Source: Front Oncol. 2020 Apr 3;10:342. doi: 10.3389/fonc.2020.00342 (PMC7145981; doi:10.3389/fonc.2020.00342)
Supplement: Supplementary file 1 [file Data_Sheet_1.PDF]

Y537S - p.Tyr537Ser – c.1610A>C

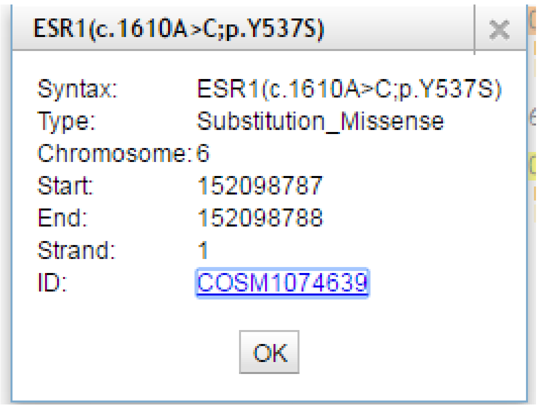

Y537N - p.Tyr537Asn - c.1609T>A

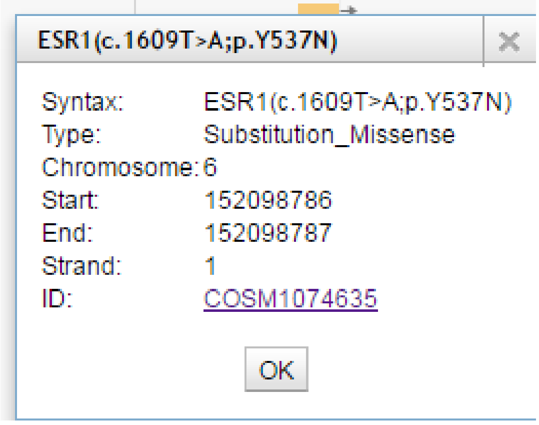

Y537C - p.Tyr537Cys - c.1610A>G (C)

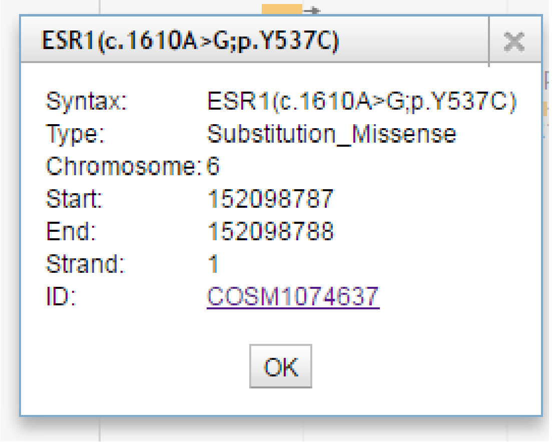

D538G - p.Asp538Gly – c.1613A>G

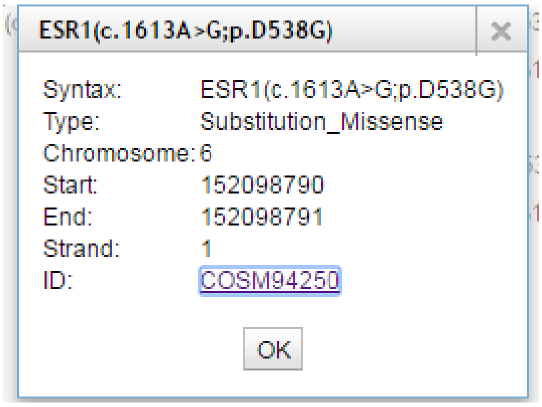

E380Q – p.Glu380Gln – c.1138G>C

```
>ESR1_ENST00000544394
GACCAGATGGTCAGTGCCTTGTTGGATGCTGAGCCCCCGATACTCTATTCCGAGTATGATCCTACCAGACCCTTCAGT
GAAGCTTCGATGATGGGCTTACTGACCAACCTGGCAGACAGGGAGCTGGTTCACATGATCAACTGGGCGAAGAGGGTG
CCAGGCTTTGTGGATTTGACCCTCCATGATCAGGTCCACCTTCTA[G/C]AATGTGCCTGGCTAGAGATCCTGATGAT
TGGTCTCGTCTGGCGCTCCATGGAGCACCCAGGGAAGCTACTGTTTGCTCCTAACTTGCTCTTGGACAGGAACCAGGG
AAAATGTGTAGAGGGCATGGTGGAGATCTTCGACATGCTGCTGGCTACATCATCTCGGTTCCGCATGATGAATCTGCA
GGGAGAGGAGTTTGTGT
```
